# Ribociclib in newly diagnosed hepatitis B infection: A case report

**DOI:** 10.3389/fonc.2023.1184952

**Published:** 2023-06-08

**Authors:** Fabrizio Di Costanzo, Simone Carrano, Gennaro Iengo, Amedeo Cefaliello, Valentina Cossiga, Filomena Morisco, Mario Giuliano, Carmine De Angelis, Grazia Arpino

**Affiliations:** ^1^ Division of Medical Oncology, Department of Clinical Medicine, University of Naples Federico II, Naples, Italy; ^2^ Department of Clinical Medicine and Surgery, Diseases of the Liver and Biliary System Unit, University of Naples “Federico II”, Naples, Italy

**Keywords:** Ribociclib, hepatitis B, luminal breast cancer, CDK4/6 inhibitors, tenofovir disoproxil fumarate

## Abstract

Breast cancer is the most frequently diagnosed cancer in women worldwide. Actually CDK4/6 inhibitor Ribociclib is approved for the treatment of metastatic hormone-positive and human epidermal growth factor receptor 2 (HER 2)-negative breast cancer, but comorbidities like infectious or cardiovascular diseases may limit its use.

**Case report:**

A 45-year-old woman was diagnosed with metastatic breast cancer in September 2021; also, her hepatitis screening resulted positive for hepatitis B infection. Patient assumed eradicative therapy for hepatitis and bit after started oncological therapy with Ribociclib.

**Outcome:**

Frequent check of hepatological function was observed since start of eradicative therapy; liver transaminases and bilirubin kept to not rise despite start of oncological treatment with Ribociclib. Patient’s Performance Status was also not compromised and revaluation at 4, 9 and 13 months showed partial response and then stable disease.

**Discussion:**

hepatotoxicity of Ribociclib is reported as a possible side effect, and often positivity for hepatitis is cause of exclusion from therapy; in our case, no hepatotoxicity was noted and patient obtained response in terms of control of both infectious and oncological diseases.

## Introduction

Female breast cancer is the most commonly diagnosed cancer worldwide; in Europe more than 400.000 women are affected every year (1), and more than 130.000 deaths due to metastatic breast cancer were reported in 2018 (2).

Prognosis and mortality are tightly linked to patient-dependent factors and to the molecular biology of the tumor itself; assessing the estrogen receptor (ER), progesterone receptor (PgR) and human epidermal growth factor receptor 2 (Erbb2, formerly HER2) expression profile is the first step to classifying the patient’s disease into prognostic and histological subtypes. The majority of patients - approximately 70% - are HR-positive and HER2-negative, with an incidence of triple positive, triple negative and HER2-enriched disease of 11%, 12% and 4% respectively (3).

The present first-line treatment involves association of CDK4/6 inhibitors and endocrine therapy as the standard of care for ER and PgR positive, HER2 negative MBC (2). Improvements in efficacy endpoints shown by these drugs were also accompanied by favorable toxicity and safety profiles, especially when compared to traditional chemotherapy (4–10); most recent data shows that, after a 53.5 median follow up, Cdk4/6 inhibitor Ribociclib is associated to significant improvements in Overall survival and Progression-free survival when administered with goserelin plus nonsteroidal aromatase inhibitor (NSAI) or tamoxifen (median OS 58.7 months with ribociclib versus 48.0 months with placebo; mPFS 27.5 months with Ribociclib versus 13.8 months, MONALEESA-7 trial (11)); Abemaciclib plus fulvestrant also prolonged Progression free survival versus placebo/fulvestrant (mPFS, 16.4 vs 9.3 months); and overall survival (OS, 46.7 vs 37.3 months; MONARCH-2 trial (12);) or when associated with NSAI (OS 67.1 months with abemaciclib plus NSAI versus 54.5 months with placebo and a NSAI; mPFS 28.2 vs 14.8 months, MONARCH-3 trial (13);). Last updates from PALOMA trial series, studying Palbociclib, seem to not show a clear advantage of the cdk4/6 inhibitor plus fulvestrant in overall survival, and the observed difference in this case was not statistically significant.

No less important, secondary publications reported that Health-related Quality of life assessment was satisfactory in patients receiving ribociclib, abemaciclib or palbociclib + ET versus placebo + ET (14–16).

Although their action and structure mechanisms are similar, differences in their toxicity profiles were nevertheless reported; Abemaciclib showed a minor rate of hematopoietic toxicity compared to Ribociclib and Palbociclib, but a major rate of diarrhea and fatigue (17, 18).

Among them, Ribociclib can induce QT prolongation and requires a periodic check of cardiac electrophysiology.

Moreover, MONALEESA series trials reported a significant rate of liver toxicity in patients treated with Ribociclib vs placebo, evidence confirmed by real life experiences; liver injury included grade 3/4 hypertransaminasemia (affecting up to 8% of patients and often enduring for many weeks despite discontinuation of therapy) (19, 20) to fulminant hepatitis (21).

This data led to their approval in combination with AI or fulvestrant in therapy for metastatic luminal breast cancer as first-line treatment or after failure of previous ET, while a first-line chemotherapy is usually reserved for patients unable to assume oral therapies or at risk of imminent organ failure (2) - though recent evidence shows relevant efficacy of cdk4/6 inhibitors even in these cases (22).

Among other patient-related prognostic factors in the treatment of MBC, infectious diseases are comorbidities that often affect treatment effectiveness and intensity; of these, one of the most common infective agents is infection with hepatitis B virus (HBV), still an important endemic infection with significant morbidity and mortality (23).

Despite vaccination programs, the spread of HBV infection and related disease is sustained by migrants and refugees with high HBsAg prevalence rates, that favor the diffusion in low endemic countries in Europe (like Italy, Germany, United Kingdom etc.) (24, 25).

In clinical practice, the presence of a preexistent unknown HBV infection or an infection not under surveillance in patients with newly diagnosed cancer is a real possibility. A recent work reports that on above 3000 newly diagnosed oncological patients screened for HBV, the observed rate for previous infection was 6.5%, and for chronic HBV 0.6% (26); an HBV screening is clearly necessary, but it can also represent cause of delay in starting oncological therapy.

In fact the prophylactic or therapeutic use of antivirals agents is able to prevent HBV replication or reactivation in the different serological categories related to HBV status during immunosuppressive or chemotherapy treatment. At moment very few data are available for patients with actively replicating HBV infection and oncological treatment; in many trial series involving cdk4/6 inhibitors their inclusion was demanded on clinician judgement (27, 28), or excluded at all (29); hence, the need to assess safety of these drugs in particular cohorts of patient, like the HBV-infected ones, whose clinical management is underreported.

## Case presentation

A 45-year-old, no smoker Caucasian woman was diagnosed with metastatic breast cancer in September 2021. In August 2021, she had undergone a right breast core biopsy, and histological examination diagnosed invasive ductal breast cancer: hormonal receptor status (ER and PgR) was positive, HER2 was not overexpressed, Ki-67 was 60%.

In September 2021, staging with 18FDG PET/CT detected breast disease, axillary and mediastinal lymph node metastases, humerus, iliac and ischium bone metastases; contrast-enhanced breast MRI and bone scintigraphy both confirmed metastatic disease.

Combination therapy with Ribociclib 600mg/die for 21 days with 28-days cycle plus Letrozole 2.5mg/day plus Triptorelin 3.75mg every four weeks was adopted as first-line treatment for this pre-menopausal, hormone receptor-positive and HER2-negative MBC. Patient had no other comorbidities and did not assume drugs before starting therapy.

Before proceeding with treatment, we evaluated infectious markers, and found hepatitis B serology positive for infection as reported below:

T0 –September 2021.
- HBsAg positive
- HBV DNA 4383 IU/mL
- HBsAb Negative
- HBcAb IgG Positive
- HBcAb IgM Negative
- HBeAg Negative
- Normal transaminases and liver function tests; no HDV coinfection.

The assessment of hepatic fibrosis by a transient elastography (fibroscan), reported a value of hepatic stiffness of 3.3 kPa and of CAP (Controlled Attenuation Parameter) of 199dB/m, indicative of absence of fibrosis and steatosis.

Following the hepatologist’s recommendations, the patient started treatment with Tenofovir disoproxil fumarate 245 mg/day for her diagnosis of hepatitis B HBeAb positive with the recommendation to check hepatitis B status (quantitative HBV-DNA) and liver function weekly, especially during the first month of treatment with Ribociclib.

**Figure 1:**
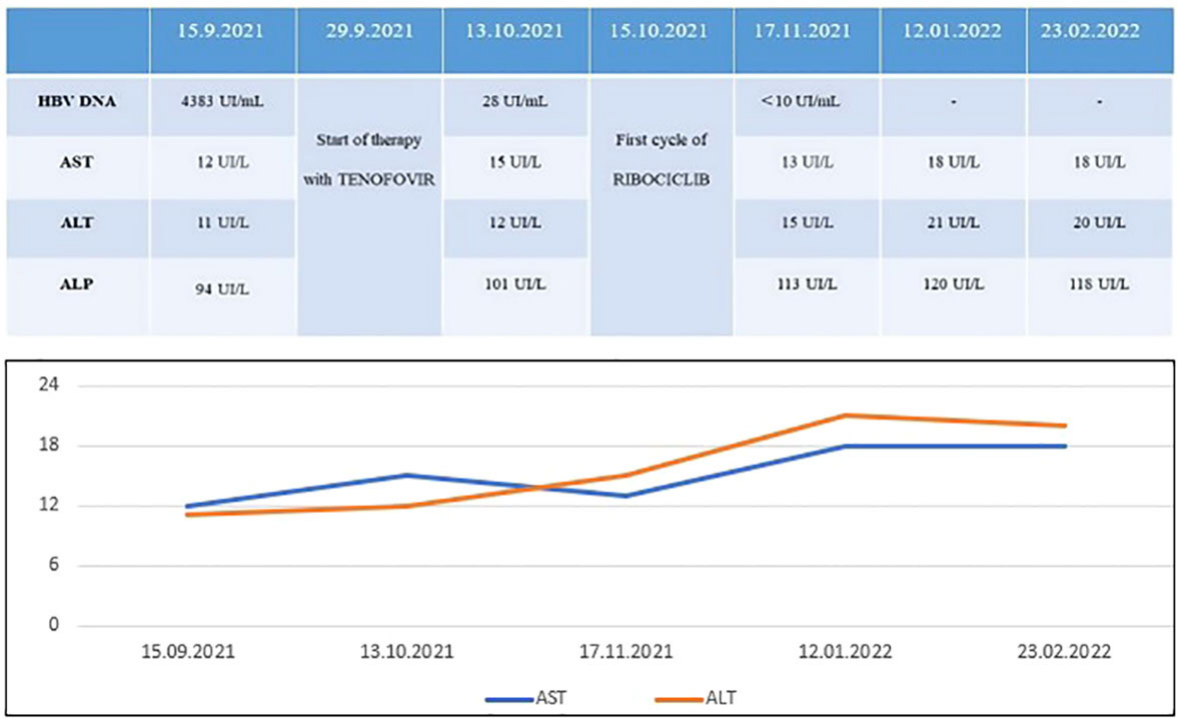
Five month-follow up of HBV DNA and liver function parameters: while HBV DNA decreased after Tenofovir, no signs of toxicity occurred after combination of Ribociclib + ET + Tenofovir.

As such, during the first cycle of treatment with Ribociclib in September 2021, we carried out weekly evaluations of HBV DNA levels, which significantly decreased (28 UI/ml) and subsequently negativized (<10 UI/ml) (**Figure 1**).

**Figure 2:**
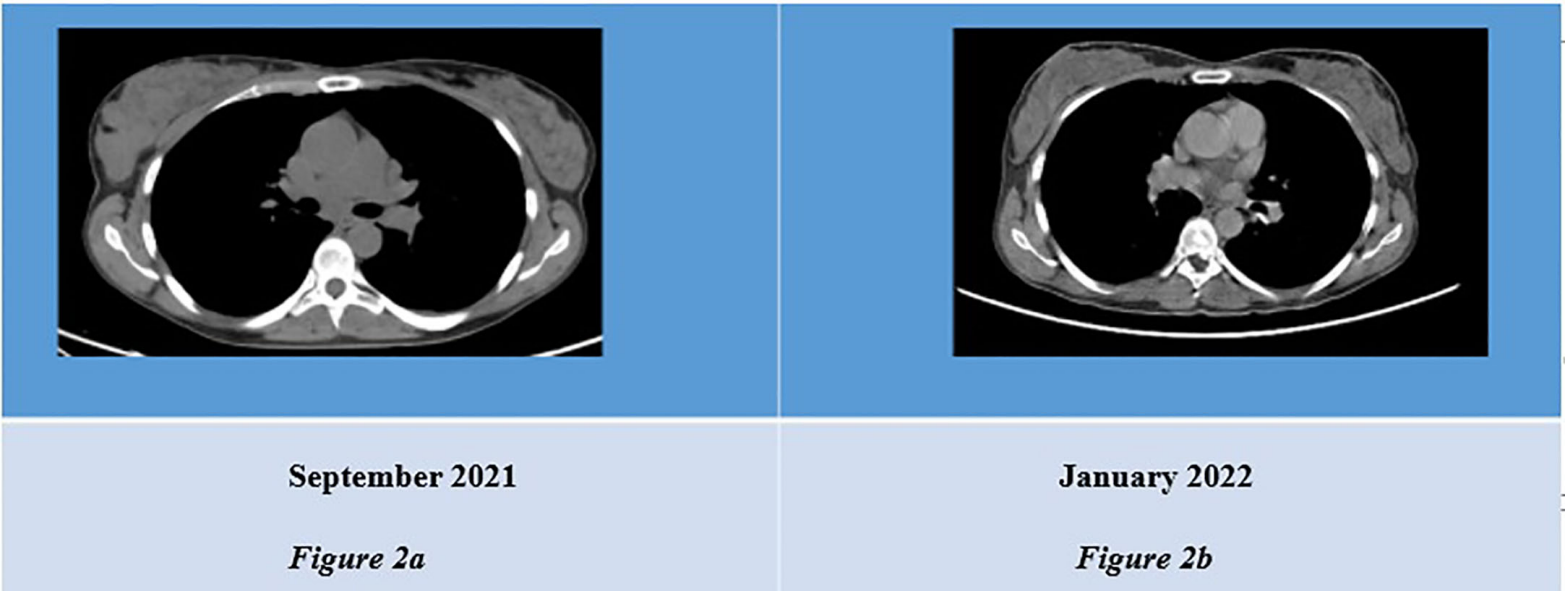
**(A)** CT scan showing thickened right mammal gland. **(B)** Revaluation CT scan showing reduced mass in infero-external right breast.

After three cycles of treatment with Ribociclib, in January 2022, 18FDG PET plus contrast-enhanced CT and breast MRI were repeated (**Figure 2**). The patient achieved a complete metabolic response and a partial response of disease (PR), according to Response Evaluation Criteria for Solid Tumors [RECIST1.1 (30)].

**Figure 3:**
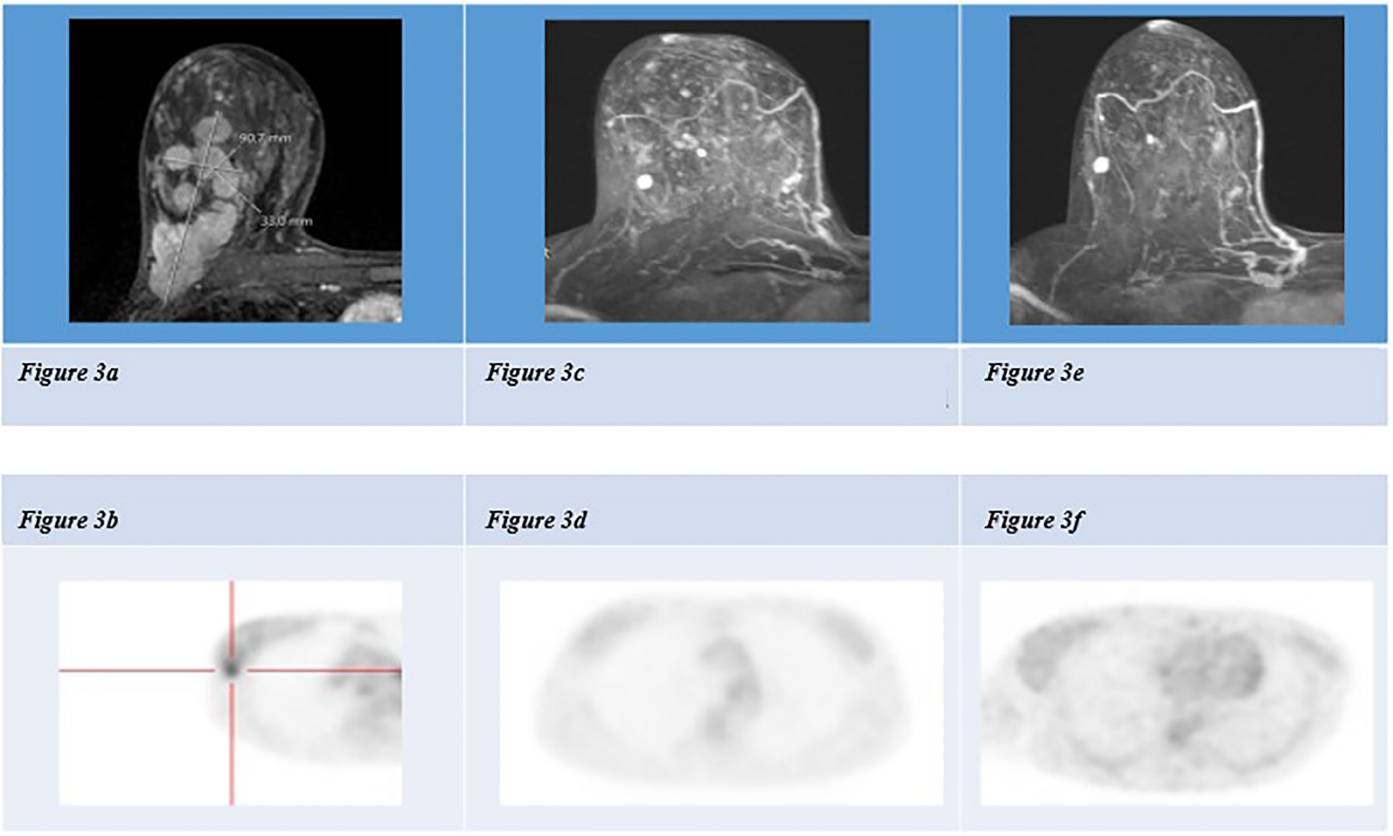
**(A)** Breast MRI of September 2021: multiple lesions occupying an area of 90x33x60mm in the right breast, below, 18-FDG PET scan of September 2021 showing contrast enhancement in right breast **(B)**. **(C)** Breast MRI of January 2022: subcutaneous nodules at infero-external and infero-internal right quadrants, with no 18- FDG uptake (see below, **D**). **(E)** Breast MRI of May 2022: subcutaneous nodules at infero- external and infero-internal right quadrants, with no 18-FDG uptake (see below, **F**).

Compared to September 2021, there were no areas of uptake at the 18FDG PET and there was a significant reduction of the breast site, lymph node and bone metastases in the contrast-enhanced CT and breast MRI; revaluations were performed in May 2022, when 18FDG PET, contrast-enhanced CT and breast MRI confirmed disease stability (**Figure 3**), and in September 2022 (stable disease).

Both times, HBV DNA levels continued to be undetectable.

**Figure 4:**
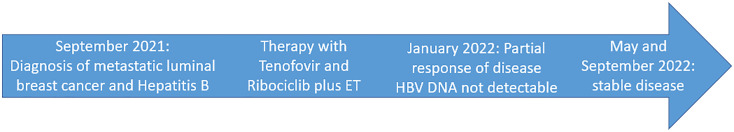
Timeline with most relevant case episodes.

Most importantly, treatment was well tolerated - with hematological toxicity not more than grade 2 according to CTCAE criteria and no need for dose reduction. No febrile neutropenia or QTc prolongation were reported; no liver toxicity emerged and the patient did not experience episodes of fatigue. Moreover, despite undergoing such an intense treatment, patient’s mood was constantly good; she did not ask or manifest need of psychological support and, to the date, patient shows a positive thinking and feelings of gratitude (**Figure 4**).

## Discussion

Female breast cancer is the leading diagnosed tumor worldwide; prognosis and treatments are related to tumor stage at time of diagnosis, and for women with non-metastatic disease (almost 65% (31, 32), therapeutic goals are tumor eradication and preventing recurrence.

Metastatic breast cancer is still an incurable disease; nevertheless, outcomes are constantly improving and new drugs are challenging this statement.

In the choice of treatment, patient status and comorbidities play a key role; infectious diseases like hepatitis often lead to the discontinuation of treatment for patients undergoing cytotoxic therapy, and many chemotherapy regimens - like anthracycline-based therapy - have been proven to cause HBV reactivation in patients with solid organ malignancies (33–35).

In our case, the first choice we had was which cdk4/6 inhibitor pick for the patient; even if a direct head-to-head comparison is not available, no clear differences in terms of efficacy between the three molecules emerge from clinical practice and clinician’s choice is usually based on patient’s age and comorbidities and on the slightly different specter of toxicities, however the switch among inhibitors is allowed if the patient develops severe side effects from one of those (20).

In our case, possible interactions with antivirals agents were a major factor to evaluate.

In order to avoid drug-induced excessive toxicities and further liver injury in the context of HBV infection, a discussion with hepatologist was hold and pharmacokinetics of all three cdk4/6 inhibitors were considered, as no clear contraindication emerged from a first analysis of literature.

Ribociclib is well known as a strong CYP3A4/5 time-dependent inhibitor, especially when administered at a 600 mg dose, and the FDA leaflet recommends to avoid the concomitant use of strong CYP3A inhibitors (e.g., clarithromycin, protease inhibitor for HIV and HCV, itraconazole, ketoconazole, posaconazole, voriconazole, ritonavir, saquinavir) because of the increase in the recorded CDKis plasma exposure that may lead to increased toxicity (36). Clinical decision about choosing Ribociclib for our patient was based on efficacy data showed by MONALEESA-7 trial, the only available study enrolling premenopausal MBC patient exclusively, and on the favorable manageability profile reported in patients with impairment of hepatic or renal function (37, 38).

As our hepatologist did not find any contraindication for use of Ribociclib in this patient and considered Tenofovir disoproxil fumarate a valid option to further protect from HBV reactivation during oncological treatment, we assessed this association as a reasonably low risk therapy for both oncological and infective diseases.

To the best of our knowledge, no case of concomitant Cdk 4/6 inhibitor + ET and anti HBV infection therapy were previously reported; the decision to treat this patient is supported by the good safety profile showed by Ribociclib both in the MONALEESA trial series and in the clinical practice and noticing the patient’s good Performance Status.

## Conclusions

We observed that it is possible to treat Hepatitis B-infection and Luminal metastatic breast cancer with both eradicative and oncological therapies; the result obtained in terms of any grade toxicity, the liver functionality remaining unaffected, the maintained response and the control over HBV infection are an encouraging outcome for treatment of patients with luminal breast cancer and hepatitis B infection.

Clearly, a risk-benefit assessment is always necessary for every patient; Authors’ proposition is that the report can be useful to clinicians when treating patients with important comorbidities like hepatitis B infection.

We also believe that this case strengthens the importance of a multidisciplinary approach. After discussion with hepatologist we were able to choose adequate therapy and, importantly, our young patient was supported from a dedicated nutritionist and, if needed, psycho-oncologist in order to fully address any potential need; this kind of integrated management allow to assess patient-tailored therapies that generally grant a prompt support and care of adverse events.

However we recognize that this integrated approach is not always feasible in all institutions and, eventually, collaboration among smaller and larger institutions should be implemented in order to deliver the same standard of care to all patients.

## Data availability statement

The original contributions presented in the study are included in the article/supplementary material. Further inquiries can be directed to the corresponding author.

## Ethics statement

Ethical review and approval was not required for the study on human participants in accordance with the local legislation and institutional requirements. The patients/participants provided their written informed consent to participate in this study. Written informed consent was obtained from the participant for the publication of this case report.

## Author contributions

All authors contributed equally to this work.
